# Crystal structure and Hirshfeld surface analysis of (2*E*)-3-(2,4-di­chloro­phen­yl)-1-(2,5-di­chloro­thio­phen-3-yl)prop-2-en-1-one

**DOI:** 10.1107/S2056989018010976

**Published:** 2018-08-10

**Authors:** T. N. Sanjeeva Murthy, Zeliha Atioğlu, Mehmet Akkurt, C. S. Chidan Kumar, M. K. Veeraiah, Ching Kheng Quah, B. P. Siddaraju

**Affiliations:** a Department of Chemistry, Sri Siddhartha Academy of Higher Education, Tumkur 572 107, Karnataka, India; b İlke Education and Health Foundation, Cappadocia University, Cappadocia Vocational College, The Medical Imaging Techniques Program, 50420 Mustafapaşa, Ürgüp, Nevşehir, Turkey; c Department of Physics, Faculty of Sciences, Erciyes University, 38039 Kayseri, Turkey; d Department of Engineering Chemistry, Vidya Vikas Institute of Engineering & Technology, Visvesvaraya Technological University, Alanahalli, Mysuru 570 028, Karnataka, India; e Department of Chemistry, Sri Siddhartha Institute of Technology, Tumkur 572 105, Karnataka, India; f X-ray Crystallography Unit, School of Physics, Universiti Sains Malaysia, 11800 USM, Penang, Malaysia; g Department of Chemistry, Cauvery Institute of Technology, Mandya 571 402, Karnataka, India

**Keywords:** crystal structure, 2,5-di­chloro­thio­phene ring, 2,4-di­chloro­phenyl ring, *E* configuration, Hirshfeld surface analysis

## Abstract

The mol­ecular structure of the title compound, C_13_H_6_Cl_4_OS, consists of a 2,5-di­chloro­thio­phene ring and a 2,4-di­chloro­phenyl ring linked *via* a prop-2-en-1-one spacer. The dihedral angle between the 2,5-di­chloro­thio­phene ring and the 2,4-di­chloro­phenyl ring is 12.24 (15)°. The mol­ecule has an *E* configuration about the C=C bond and the carbonyl group is *syn* with respect to the C=C bond. The mol­ecular conformation is stabilized by intra­molecular C—H⋯Cl contacts, producing *S*(6) and *S*(5) ring motifs. In the crystal, the mol­ecules are linked along the *a-*axis direction through face-to-face π-stacking between the thio­phene rings and the benzene rings of the mol­ecules in zigzag sheets lying parallel to the *bc* plane along the *c* axis. The inter­molecular inter­actions in the crystal packing were further analysed using Hirshfield surface analysis, which indicates that the most significant contacts are Cl⋯H/ H⋯Cl (20.8%), followed by Cl⋯Cl (18.7%), C⋯C (11.9%), Cl⋯S/S⋯Cl (10.9%), H⋯H (10.1%), C⋯H/H⋯C (9.3%) and O⋯H/H⋯O (7.6%).

## Abstract 2

In the title compound, the 2,5-di­chloro­thio­phene and 2,4-di­chloro­phenyl rings, linked *via* a prop-2-en-1-one spacer, make a dihedral angle of 12.24 (15)°. Both the thio­phene and benzene rings of adjacent mol­ecules inter­act attractively in a face-to-face manner, forming zigzag sheets lying parallel to the (011) plane.

## Chemical context   

Compounds bearing the 1,3-diphenyl-2-propen-1-one framework and belong to the flavonoid family are commonly called by its generic name ‘chalcone’. These are abundant in nature, ranging from ferns to higher plants, and are considered to be the precursors of flavonoids and isoflavonoids, in which the two aromatic rings are joined by a three carbon α,β-unsaturated carbonyl system. In plants, chalcones are converted to the corresponding (2*S*)-flavanones in a stereospecific reaction catalysed by the enzyme chalcone isomerase. The chemistry of chalcones remains a fascination among researchers because of the large number of replaceable hydrogen atoms that allows a number of derivatives with a variety of promising biological activities. They are found in fruits and vegetables, which attracted attention because of their pharmacological activities such as anti-inflamatory (Yadav *et al.*, 2011 ▸), anti­fungal (Mahapatra *et al.*, 2015 ▸), anti­viral (Nowakowska, 2007 ▸; Chimenti *et al.*, 2010 ▸; Elarfi &Al-Difar, 2012 ▸), anti­oxidant (Ferreira *et al.*, 2006 ▸) and anti­cancer (Stiborova *et al.*, 2011 ▸ activities). The synthesis and anti­microbial evaluation of new chalcones containing a 2,5-di­chloro­thio­phene moiety has been reported (Tomar *et al.*, 2007 ▸). In recent years, chalcones have been used in the field of materials science as non-linear optical devices (Raghavendra *et al.*, 2017 ▸; Chandra Shekhara Shetty *et al.*, 2016 ▸). In view of all the above and as part of our ongoing work (Harrison *et al.*, 2010 ▸; Jasinski *et al.*, 2010 ▸; Dutkiewicz *et al.*, 2010 ▸) herewith we report the crystal and mol­ecular structure of the title compound.
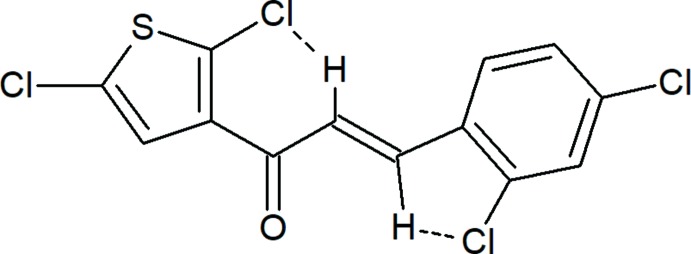


## Structural commentary   

**Figure 1:**
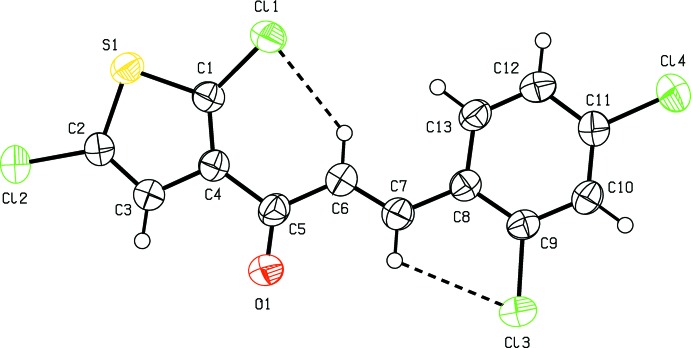
The mol­ecular structure of the title compound, showing the atom labelling and displacement ellipsoids drawn at the 50% probability level. The two intra­molecular C—H⋯Cl contacts (see Table1) are shown as dashed lines.

The title compound, Fig. 1 ▸, is constructed from two aromatic rings (2,5-di­chloro­thio­phene and terminal 2,4-di­chloro­phenyl rings), which are linked by a C=C—C(=O)—C enone bridge. Probably as a result of the steric repulsion between the chlorine atoms of the adjacent mol­ecules, the C3—C4—C5—O1 and O1—C5—C6—C7 torsion angles about the enone bridge are −11.8 (5) and 0.4 (6)°, respectively. Hence, the dihedral angle between the 2,5-di­chloro­thio­phene ring and the 2,4-di­chloro­phenyl ring increases to 12.24 (15)°. The bond lengths and angles in the title compound are comparable with those of the related compounds (*E*)-3-(3,4-di­meth­oxy­phen­yl)-1-(1-hy­droxy­naphthalen-2­yl)prop-2-en-1-one (Ezhilarasi *et al.*, 2015 ▸), (*E*)-1-(3-bromo­phen­yl)-3-(3,4-di­meth­oxy­phen­yl)prop-2-en-1-one (Escobar *et al.*, 2012 ▸) and (*E*)-3-(2-bromo­phen­yl)-1-(3,4-di­meth­oxy­phen­yl)prop-2-en-1-one (Li *et al.*, 2012 ▸). The mol­ecular conformation of the title compound is stabilized by intra­molecular C—H⋯Cl contacts (Table 1 ▸), producing *S*(6) and *S*(5) ring motifs.

## Supra­molecular features and Hirshfeld surface analysis   

**Figure 2:**
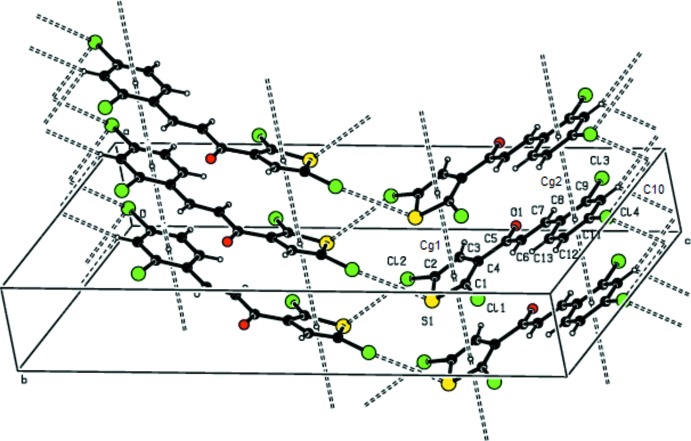
A view of the offset face-to-face π-stacking in the title compound, with the thick dashed lines indicating centroid-to-centroid inter­actions. The Cl⋯H and Cl⋯S inter­actions are also shown as dashed lines.

In the crystal, conventional hydrogen bonds are not observed. π-stacking is observed between the thio­phene rings (S1/C1–C4, centroid *Cg*1) of adjacent mol­ecules in the alternating sheets along the [100] direction [*Cg*1⋯*Cg*1^i,ii^: centroid–centroid distance = 3.987 (2) Å, shortest perpendicular distance for the centroid of one ring to the plane of the other = 3.6143 (12) Å, ring-centroid offset = 1.683 Å; symmetry codes: (i) −1 + *x*, *y*, *z*; (i) 1 + *x*, *y*, *z*] and between the benzene rings (C8–C13, centroid *Cg*2) of the same mol­ecules [*Cg*2⋯*Cg*2^i,ii^: centroid–centroid distance = 3.987 (2) Å, shortest perpendic­ular distance = 3.5213 (13) Å, offset = 1.869 Å]. As shown Fig. 2 ▸, the mol­ecules are packed to form zigzag sheets lying parallel to (011) along the *c*-axis direction through face-to-face π-stacking between the thio­phene and benzene rings of pairs of adjacent mol­ecules along the [100] direction (Cl⋯S and Cl⋯H inter­actions; Table 2 ▸ and Fig. 2 ▸). The Cl⋯S contact, at 3.660 (1) Å, is equal to the sum of the van der Waals radii of S and Cl atoms (3.65 Å; Pauling, 1960 ▸).

**Figure 3:**
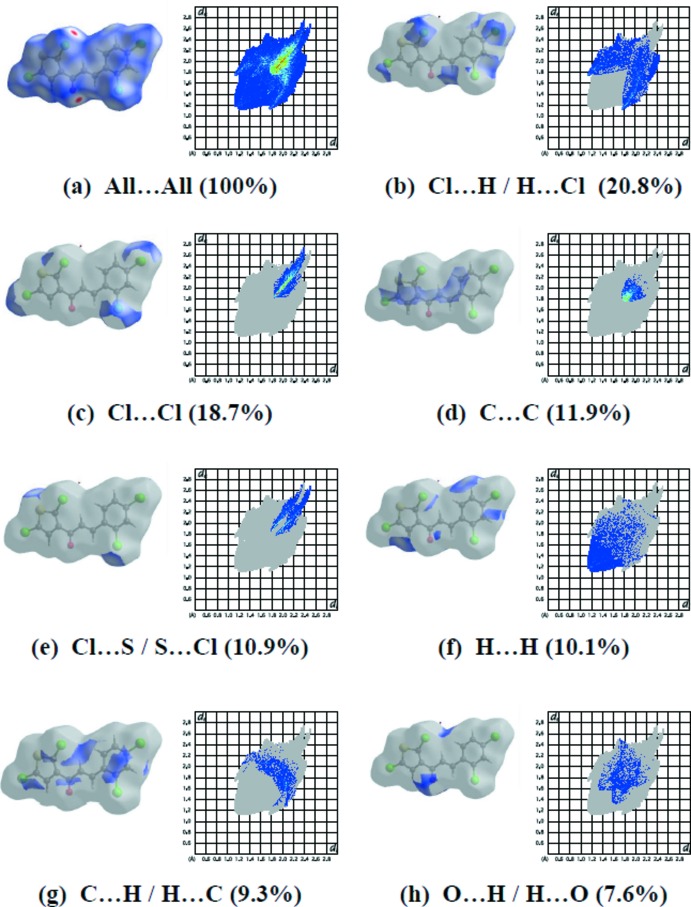
The two-dimensional fingerprint plots of the title compound, showing (*a*) all inter­actions, and delineated into (*b*) Cl⋯H/ H⋯Cl, (*c*) Cl⋯Cl, (*d*) C⋯C, (*e*) Cl⋯S/S⋯Cl, (*f*) H⋯H, (*g*) C⋯H/H⋯C and (*h*) O⋯H/H⋯O inter­actions.

Hirshfeld surfaces and fingerprint plots were generated for the title compound using *CrystalExplorer* (McKinnon *et al.*, 2007 ▸). Hirshfeld surfaces enable the visualization of inter­molecular inter­actions by different colours and colour intensity, representing short or long contacts and indicating the relative strength of the inter­actions. The overall two-dimensional fingerprint plot for the title compound and those delineated into Cl⋯H/ H⋯Cl, Cl⋯Cl, C⋯C, Cl⋯S/S⋯Cl, H⋯H, C⋯H/H⋯C and O⋯H/H⋯O contacts are illus­trated in Fig. 3 ▸; the percentage contributions from the different inter­atomic contacts to the Hirshfeld surfaces are as follows: Cl⋯H/ H⋯Cl (20.8%), Cl⋯Cl (18.7%), C⋯C (11.9%), Cl⋯S/S⋯Cl (10.9%), H⋯H (10.1%), C⋯H/H⋯C (9.3%) and O⋯H/H⋯O (7.6%). The contributions of the other weak inter­molecular contacts to the Hirshfeld surfaces are Cl⋯C/C⋯Cl (3.6%), S⋯C/C⋯S (2.8%), Cl⋯O/O⋯Cl (2.3%), S⋯S (0.9%), O⋯O (0.6%) and C⋯O/O⋯C (0.6%).

The C—H⋯Cl inter­actions appear as two distinct spikes in the fingerprint plot (Fig. 3 ▸ *b*) of the title compound, where the sum of Cl⋯H/H⋯Cl inter­actions comprises 20.8% of the total Hirshfeld surface area of the mol­ecule. The Cl⋯H/H⋯Cl inter­actions represented by the spikes in the bottom right and left region (*d*_e_ + *d*_i_ ≃ 2.83 Å) indicate that the hydrogen atoms are in contact with the Cl atoms to build the two-dimensional supra­molecular framework [*d*_e_ and *d*_i_ represent the distances from a point on the Hirshfeld surface to the nearest atoms outside (external) and inside (inter­nal) the surface, respectively]. Cl⋯Cl contacts (Fig. 3 ▸ *c*; 18.7%) are disfavoured when the number of H atoms on the mol­ecular surface is large because of competition with the more attractive H⋯Cl contacts. Cl⋯Cl contacts from a parallel alignment of C—Cl bonds (C10—H10*A*⋯Cl4^iii^; (iii) −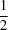 + *x*, 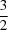 − *y*, 1 − *z*] may be indicated. They are known in the literature as type-I halogen–halogen inter­actions (Bui *et al.*, 2009 ▸), with both C—Cl⋯Cl angles equal to one another. In the present case, these angles are close to 165°. The C⋯C contacts (Fig. 3 ▸ *d*); 11.9%) reflect π–π inter­actions between the above-mentioned aromatic rings. The S⋯Cl contacts (Fig. 3 ▸ *e*; 10.9%) contracted to a much lesser degree. The C⋯H/H⋯C inter­actions (Fig. 3 ▸ *g*) account for 9.3% of the total Hirshfeld surface of the mol­ecules. The scattered points in the breakdown of the fingerprint plot show the π–π stacking inter­actions. In the fingerprint plot delineated into H⋯O/O⋯H contacts (Fig. 3 ▸ *h*), the 7.6% contribution to the Hirshfeld surface arises from inter­molecular C=O⋯H hydrogen bonding and is viewed as pair of spikes with the tip at *de* + *d*i ∼ 2.9 Å.

The large number of Cl⋯H/ H⋯Cl, Cl⋯Cl, C⋯C, Cl⋯S/S⋯Cl, H⋯H, C⋯H/H⋯C and O⋯H/H⋯O inter­actions suggest that van der Waals inter­actions and hydrogen bonding play the major roles in the crystal packing (Hathwar *et al.*, 2015 ▸).

## Database survey   

The closest related compounds with the same skeleton and containing a similar bis-chalcone moiety to the title compound but with different substituents on the aromatic rings are: (2*E*)-1-(5-chloro­thio­phen-2-yl)-3-(4-ethyl­phen­yl)prop-2-en-1-one [(I); Naik *et al.*, 2015 ▸], (2*E*)-1-(5-bromo­thio­phen-2-yl)-3-(4-ethyl­phen­yl)prop- 2-en-1-one [(II); Naik *et al.*, 2015 ▸], (2*E*)-1-(5-chloro­thio­phen-2-yl)-3-(4-eth­oxy­phen­yl)prop-2-en-1-one [(III); Naik *et al.*, 2015 ▸], (2*E*)-1-(5-bromo­thio­phen-2-yl)-3-(4-eth­oxy­phen­yl)prop-2-en-1-one [(IV); Naik *et al.*, 2015 ▸], (2*E*)-3-(4-bromo­phen­yl)-1-(5-chloro­thio­phen-2-yl)prop-2-en-1-one [(V); Naik *et al.*, 2015 ▸], (2*E*)-1-(5-bromo­thio­phen-2-yl)-3-(3-meth­oxy­phen­yl)prop-2-en-1-one [(VI); Naik *et al.*, 2015 ▸], (*E*)-1-(5-chloro­thio­phen-2-yl)-3-(p-tol­yl)prop-2-en-1-one [(VII); Kumara *et al.*, 2017 ▸], (*E*)-1-(5-chloro­thio­phen-2-yl)-3-(2,4-di­methyl­phen­yl) prop-2-en-1-one [(VIII); Naveen *et al.*, 2016 ▸], (2*E*)-1-(5-bromo­thio­phen- 2-yl)-3-(2-chloro­phen­yl)prop-2-en-1-one [(IX); Anitha *et al.*, 2015 ▸], (2*E*)-1-[4-hy­droxy-3-(morpholin-4-ylmeth­yl)phen­yl]-3-(thio­phen-2-yl)prop-2-en-1-one [(X); Yesilyurt *et al.*, 2018 ▸] and (*E*)-1-(2-amino­phen­yl)-3-(thio­phen-2-yl)prop-2-en-1-one [(XI); Chantrapromma *et al.*, 2013 ▸].

In (I) and (II), the structures are isostructural in space group *P*1, while (III) and (IV) are isostructural in space group *P*2_1_/*c*. There are no hydrogen bonds of any kind in the structures of compounds (I) and (II), but in the structures of compounds (III) and (IV), the mol­ecules are linked into *C*(7) chains by means of C—H⋯O hydrogen bonds. In (V), there are again no hydrogen bonds nor π–π stacking inter­actions but in (VI), the mol­ecules are linked into *C*(5) chains by C—H⋯O hydrogen bonds. In each of compounds (I)–(VI), the mol­ecular skeletons are close to planarity, and there are short halogen–halogen contacts in the structures of compounds (II) and (V) and a short Br⋯O contact in the structure of compound (VI).

In (VII), the mol­ecule is non-planar, with a dihedral angle of 22.6 (2)° between the aromatic rings. The mol­ecules are linked by pairs of C—H⋯π inter­actions, forming inversion dimers. There are no other significant inter­molecular inter­actions present. In (VIII), the mol­ecule is nearly planar, the dihedral angle between the thio­phene and phenyl rings being 9.07 (8)°. The mol­ecules are linked *via* weak C—H⋯O and C—H⋯S hydrogen bonds, forming chains propagating along the *c-*axis direction. In (IX), the thienyl ring is not coplanar with the benzene ring, their planes forming a dihedral angle of 13.2 (4)°. In the crystal, mol­ecules stack along the *a-*axis direction, with the inter­planar separation between the thienyl rings and between the benzene rings being 3.925 (6) Å. In (X), the thio­phene ring forms a dihedral angle of 26.04 (9)° with the benzene ring. The mol­ecular conformation is stabilized by an O—H⋯N hydrogen bond. The mol­ecules are connected through C—H⋯O hydrogen bonds, forming wave-like layers parallel to the *ab* plane, which are further linked into a three-dimensional network by C—H⋯π inter­actions. In (XI), the mol­ecule is almost planar with a dihedral angle of 3.73 (8)° between the phenyl and thio­phene rings. An intra­molecular N—H⋯O hydrogen bond generates an *S*(6) ring motif. Adjacent mol­ecules are linked into dimers in an anti-parallel face-to-face manner by pairs of C—H⋯O inter­actions. Neighboring dimers are further linked into chains along the *c*-axis direction by N—H⋯N hydrogen bonds.

## Synthesis and crystallization   

The title compound was synthesized as per the procedure reported earlier (Kumar *et al.*, 2013*a* ▸,*b* ▸; Chidan Kumar *et al.*, 2014 ▸). 1-(2,5-Di­chloro­thio­phen-3-yl)ethanone (0.01 mol) (Harrison *et al.*, 2010 ▸) and 2,4-di­chloro­benzaldehyde (0.01 mol) was dissolved in 20 ml methanol. A catalytic amount of NaOH was added to the solution dropwise with vigorous stirring. The reaction mixture was stirred for about 2 h at room temperature. The formed crude products were filtered, washed successively with distilled water and recrystallized from methanol to get the title chalcone. The melting point (381–383 K) was determined by Stuart Scientific (UK) apparatus.

## Refinement   

Crystal data, data collection and structure refinement details are summarized in Table 3 ▸. C-bound H atoms were positioned geometrically and refined using a riding model, with C—H = 0.93 Å and *U*_iso_(H) = 1.2*U*_eq_(C) for C—H. Owing to poor agreement between observed and calculated intensities, twelve outliers (2 7 2, 2 8 0, 2 8 1, 0 1 28, 2 8 23, 0 14 8, 0 0 6, 3 0 29, 1 0 8, 0 17 4, 1 3 27, 2 12 19) were omitted in the final cycles of refinement.

## Supplementary Material

Crystal structure: contains datablock(s) I, global. DOI: 10.1107/S2056989018010976/dx2006sup1.cif

Structure factors: contains datablock(s) I. DOI: 10.1107/S2056989018010976/dx2006Isup2.hkl

Supporting information file. DOI: 10.1107/S2056989018010976/dx2006Isup3.cml

CCDC reference: 1036797

Additional supporting information:  crystallographic information; 3D view; checkCIF report

## Figures and Tables

**Table 1:** Hydrogen-bond geometry (Å, °)

*D*—H⋯*A*	*D*—H	H⋯*A*	*D*⋯*A*	*D*—H⋯*A*
C6—H6*A*⋯Cl1	0.93	2.48	3.220 (3)	136
C7—H7*A*⋯Cl3	0.93	2.65	3.075 (3)	108

**Table 2:** Summary of short inter­atomic contacts (Å) in the title compound

Contact	Distance	Symmetry operation
Cl2⋯S1	3.660 (1)	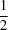 + *x*, 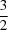 − *y*, 2 − *z*
H10*A*⋯Cl4	3.03	− 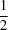 + *x*, 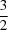 − *y*, 1 − *z*
C8⋯C9	3.573 (4)	1 + *x*, *y*, *z*

**Table 3:** Experimental details

Crystal data	
Chemical formula	C_13_H_6_Cl_4_OS
*M*_r_	352.04
Crystal system, space group	Orthorhombic, *P*2_1_2_1_2_1_
Temperature (K)	294
*a*, *b*, *c* (Å)	3.9867 (3), 13.4564 (11), 25.573 (2)
*V* (Å^3^)	1371.91 (19)
*Z*	4
Radiation type	Mo *K*α
μ (mm^−1^)	1.00
Crystal size (mm)	0.63 × 0.23 × 0.11

Data collection	
Diffractometer	Bruker APEXII CCD
Absorption correction	Multi-scan (*SADABS*; Bruker, 2007 ▸)
*T*_min_, *T*_max_	0.757, 0.894
No. of measured, independent and observed [*I* > 2σ(*I*)] reflections	11402, 4226, 3425
*R*_int_	0.026
(sin θ/λ)_max_ (Å^−1^)	0.720

Refinement	
*R*[*F* ^2^ > 2σ(*F* ^2^)], *wR*(*F* ^2^), *S*	0.038, 0.102, 1.03
No. of reflections	4226
No. of parameters	172
H-atom treatment	H-atom parameters constrained
Δρ_max_, Δρ_min_ (e Å^−3^)	0.25, −0.20
Absolute structure	Flack *x* determined using 1124 quotients [(*I* ^+^)−(*I* ^−^)]/[(*I* ^+^)+(*I* ^−^)] (Parsons *et al.*, 2013 ▸)
Absolute structure parameter	0.04 (5)

Computer programs: *APEX2* and *SAINT* (Bruker, 2007 ▸), *SHELXS97* (Sheldrick, 2008 ▸), *SHELXL2014* (Sheldrick, 2015 ▸), *ORTEP-3 for Windows* (Farrugia, 2012 ▸) and *PLATON* (Spek, 2009 ▸).

## supplementary crystallographic information

### Crystal data

**Table:**

C_13_H_6_Cl_4_OS	*D*_x_ = 1.704 Mg m^−^^3^
*M**_r_* = 352.04	Mo *K*α radiation, λ = 0.71073 Å
Orthorhombic, *P*2_1_2_1_2_1_	Cell parameters from 4362 reflections
*a* = 3.9867 (3) Å	θ = 2.2–28.5°
*b* = 13.4564 (11) Å	µ = 1.00 mm^−^^1^
*c* = 25.573 (2) Å	*T* = 294 K
*V* = 1371.91 (19) Å^3^	Block, yellow
*Z* = 4	0.63 × 0.23 × 0.11 mm
*F*(000) = 704	

### Data collection

**Table:**

Bruker APEXII CCD diffractometer	3425 reflections with *I* > 2σ(*I*)
φ and ω scans	*R*_int_ = 0.026
Absorption correction: multi-scan (SADABS; Bruker, 2007)	θ_max_ = 30.8°, θ_min_ = 1.6°
*T*_min_ = 0.757, *T*_max_ = 0.894	*h* = −5→2
11402 measured reflections	*k* = −19→19
4226 independent reflections	*l* = −36→36

### Refinement

**Table:**

Refinement on *F*^2^	Hydrogen site location: inferred from neighbouring sites
Least-squares matrix: full	H-atom parameters constrained
*R*[*F*^2^ > 2σ(*F*^2^)] = 0.038	*w* = 1/[σ^2^(*F*_o_^2^) + (0.0581*P*)^2^ + 0.011*P*] where *P* = (*F*_o_^2^ + 2*F*_c_^2^)/3
*wR*(*F*^2^) = 0.102	(Δ/σ)_max_ = 0.001
*S* = 1.03	Δρ_max_ = 0.25 e Å^−^^3^
4226 reflections	Δρ_min_ = −0.20 e Å^−^^3^
172 parameters	Absolute structure: Flack *x* determined using 1124 quotients [(*I*^+^)-(*I*^-^)]/[(*I*^+^)+(*I*^-^)] (Parsons *et al.*, 2013)
0 restraints	Absolute structure parameter: 0.04 (5)

### Special details

**Table:**

Geometry. All esds (except the esd in the dihedral angle between two l.s. planes) are estimated using the full covariance matrix. The cell esds are taken into account individually in the estimation of esds in distances, angles and torsion angles; correlations between esds in cell parameters are only used when they are defined by crystal symmetry. An approximate (isotropic) treatment of cell esds is used for estimating esds involving l.s. planes.

### Fractional atomic coordinates and isotropic or equivalent isotropic displacement parameters (Å^2^)

**Table:**

	*x*	*y*	*z*	*U*_iso_*/*U*_eq_	
C1	1.1673 (8)	0.77797 (19)	0.84012 (9)	0.0391 (6)	
C2	1.2553 (8)	0.6794 (2)	0.91880 (10)	0.0419 (6)	
C3	1.1115 (8)	0.6258 (2)	0.88062 (10)	0.0410 (6)	
H3A	1.052432	0.559246	0.884223	0.049*	
C4	1.0587 (8)	0.6820 (2)	0.83366 (10)	0.0382 (6)	
C5	0.9016 (9)	0.6327 (2)	0.78763 (10)	0.0444 (7)	
C6	0.7779 (10)	0.6938 (2)	0.74420 (11)	0.0493 (7)	
H6A	0.809837	0.762158	0.746266	0.059*	
C7	0.6253 (9)	0.6588 (2)	0.70264 (10)	0.0462 (7)	
H7A	0.596005	0.590316	0.700760	0.055*	
C8	0.4975 (8)	0.7177 (2)	0.65917 (10)	0.0386 (6)	
C9	0.3384 (8)	0.67552 (19)	0.61621 (10)	0.0403 (6)	
C10	0.2191 (8)	0.7316 (2)	0.57472 (10)	0.0431 (6)	
H10A	0.112503	0.701315	0.546561	0.052*	
C11	0.2620 (8)	0.8330 (2)	0.57612 (10)	0.0425 (7)	
C12	0.4192 (9)	0.8788 (2)	0.61805 (11)	0.0465 (7)	
H12A	0.447771	0.947373	0.618483	0.056*	
C13	0.5316 (9)	0.8219 (2)	0.65879 (11)	0.0438 (7)	
H13A	0.633719	0.852975	0.687101	0.053*	
O1	0.8718 (9)	0.54311 (16)	0.78790 (9)	0.0721 (9)	
S1	1.3313 (2)	0.80047 (5)	0.90119 (3)	0.04511 (19)	
Cl1	1.1738 (3)	0.87633 (5)	0.79734 (3)	0.0556 (2)	
Cl2	1.3606 (3)	0.63887 (6)	0.98017 (3)	0.0593 (2)	
Cl3	0.2772 (3)	0.54840 (5)	0.61241 (3)	0.0639 (3)	
Cl4	0.1204 (3)	0.90504 (6)	0.52453 (3)	0.0605 (2)	

### Atomic displacement parameters (Å^2^)

**Table:**

	*U*^11^	*U*^22^	*U*^33^	*U*^12^	*U*^13^	*U*^23^
C1	0.0422 (17)	0.0375 (11)	0.0377 (11)	0.0021 (13)	0.0062 (12)	−0.0015 (9)
C2	0.0429 (18)	0.0443 (13)	0.0384 (12)	0.0015 (13)	−0.0020 (11)	0.0019 (10)
C3	0.0430 (18)	0.0392 (12)	0.0408 (12)	−0.0001 (13)	−0.0012 (12)	0.0003 (10)
C4	0.0376 (16)	0.0406 (12)	0.0364 (11)	0.0014 (12)	0.0021 (11)	−0.0021 (10)
C5	0.051 (2)	0.0462 (14)	0.0362 (12)	−0.0046 (14)	0.0005 (12)	−0.0046 (10)
C6	0.059 (2)	0.0451 (13)	0.0437 (13)	−0.0026 (15)	−0.0080 (14)	−0.0013 (11)
C7	0.058 (2)	0.0429 (13)	0.0382 (12)	−0.0006 (15)	0.0010 (14)	−0.0022 (10)
C8	0.0385 (16)	0.0415 (13)	0.0358 (11)	0.0001 (12)	0.0045 (11)	−0.0038 (10)
C9	0.0416 (16)	0.0380 (11)	0.0412 (12)	−0.0018 (13)	0.0027 (13)	−0.0046 (9)
C10	0.0433 (18)	0.0481 (13)	0.0378 (12)	0.0008 (13)	0.0001 (12)	−0.0066 (10)
C11	0.0387 (18)	0.0488 (14)	0.0401 (12)	0.0061 (13)	0.0018 (11)	0.0002 (10)
C12	0.048 (2)	0.0396 (13)	0.0522 (15)	−0.0007 (13)	0.0013 (14)	−0.0061 (11)
C13	0.0468 (19)	0.0422 (13)	0.0426 (13)	−0.0004 (13)	−0.0034 (13)	−0.0080 (11)
O1	0.123 (3)	0.0414 (11)	0.0517 (12)	−0.0110 (15)	−0.0209 (16)	−0.0012 (9)
S1	0.0504 (5)	0.0422 (3)	0.0427 (3)	−0.0035 (3)	−0.0008 (3)	−0.0055 (3)
Cl1	0.0766 (6)	0.0403 (3)	0.0498 (4)	−0.0043 (4)	−0.0001 (4)	0.0048 (3)
Cl2	0.0740 (6)	0.0589 (4)	0.0450 (3)	−0.0016 (4)	−0.0159 (4)	0.0056 (3)
Cl3	0.0883 (8)	0.0410 (3)	0.0625 (4)	−0.0127 (4)	−0.0148 (5)	−0.0023 (3)
Cl4	0.0684 (6)	0.0556 (4)	0.0576 (4)	0.0076 (4)	−0.0106 (4)	0.0080 (3)

### Geometric parameters (Å, º)

**Table:**

C1—C4	1.372 (4)	C7—C8	1.458 (4)
C1—Cl1	1.717 (3)	C7—H7A	0.9300
C1—S1	1.720 (3)	C8—C9	1.390 (4)
C2—C3	1.343 (4)	C8—C13	1.408 (4)
C2—Cl2	1.714 (3)	C9—C10	1.386 (4)
C2—S1	1.717 (3)	C9—Cl3	1.731 (3)
C3—C4	1.435 (4)	C10—C11	1.375 (4)
C3—H3A	0.9300	C10—H10A	0.9300
C4—C5	1.489 (4)	C11—C12	1.387 (4)
C5—O1	1.212 (4)	C11—Cl4	1.732 (3)
C5—C6	1.467 (4)	C12—C13	1.368 (4)
C6—C7	1.312 (4)	C12—H12A	0.9300
C6—H6A	0.9300	C13—H13A	0.9300
			
C4—C1—Cl1	130.8 (2)	C8—C7—H7A	117.1
C4—C1—S1	113.3 (2)	C9—C8—C13	116.5 (3)
Cl1—C1—S1	115.92 (16)	C9—C8—C7	122.7 (3)
C3—C2—Cl2	126.8 (2)	C13—C8—C7	120.9 (3)
C3—C2—S1	113.3 (2)	C10—C9—C8	122.6 (3)
Cl2—C2—S1	119.95 (17)	C10—C9—Cl3	116.5 (2)
C2—C3—C4	112.8 (3)	C8—C9—Cl3	120.8 (2)
C2—C3—H3A	123.6	C11—C10—C9	118.5 (3)
C4—C3—H3A	123.6	C11—C10—H10A	120.7
C1—C4—C3	110.5 (2)	C9—C10—H10A	120.7
C1—C4—C5	130.3 (2)	C10—C11—C12	121.2 (3)
C3—C4—C5	119.2 (3)	C10—C11—Cl4	119.7 (2)
O1—C5—C6	121.9 (3)	C12—C11—Cl4	119.2 (2)
O1—C5—C4	118.7 (3)	C13—C12—C11	119.2 (3)
C6—C5—C4	119.3 (3)	C13—C12—H12A	120.4
C7—C6—C5	124.6 (3)	C11—C12—H12A	120.4
C7—C6—H6A	117.7	C12—C13—C8	122.0 (3)
C5—C6—H6A	117.7	C12—C13—H13A	119.0
C6—C7—C8	125.7 (3)	C8—C13—H13A	119.0
C6—C7—H7A	117.1	C2—S1—C1	90.24 (13)
			
Cl2—C2—C3—C4	−179.6 (2)	C13—C8—C9—C10	0.3 (5)
S1—C2—C3—C4	0.7 (4)	C7—C8—C9—C10	−179.5 (3)
Cl1—C1—C4—C3	178.6 (3)	C13—C8—C9—Cl3	−179.3 (3)
S1—C1—C4—C3	0.2 (4)	C7—C8—C9—Cl3	0.9 (4)
Cl1—C1—C4—C5	−2.0 (6)	C8—C9—C10—C11	0.4 (5)
S1—C1—C4—C5	179.6 (3)	Cl3—C9—C10—C11	179.9 (3)
C2—C3—C4—C1	−0.6 (4)	C9—C10—C11—C12	−0.3 (5)
C2—C3—C4—C5	179.9 (3)	C9—C10—C11—Cl4	179.2 (2)
C1—C4—C5—O1	168.9 (4)	C10—C11—C12—C13	−0.3 (5)
C3—C4—C5—O1	−11.8 (5)	Cl4—C11—C12—C13	−179.9 (3)
C1—C4—C5—C6	−13.1 (5)	C11—C12—C13—C8	1.0 (5)
C3—C4—C5—C6	166.3 (3)	C9—C8—C13—C12	−1.0 (5)
O1—C5—C6—C7	0.4 (6)	C7—C8—C13—C12	178.8 (3)
C4—C5—C6—C7	−177.6 (3)	C3—C2—S1—C1	−0.5 (3)
C5—C6—C7—C8	179.5 (3)	Cl2—C2—S1—C1	179.8 (2)
C6—C7—C8—C9	179.9 (4)	C4—C1—S1—C2	0.1 (3)
C6—C7—C8—C13	0.1 (5)	Cl1—C1—S1—C2	−178.5 (2)

### Hydrogen-bond geometry (Å, º)

**Table:**

*D*—H···*A*	*D*—H	H···*A*	*D*···*A*	*D*—H···*A*
C6—H6*A*···Cl1	0.93	2.48	3.220 (3)	136
C7—H7*A*···Cl3	0.93	2.65	3.075 (3)	108
